# DECAF versus CURB-65 to Foresee Mortality among Patients Presenting with an Acute Exacerbation of Chronic Obstructive Pulmonary Disease

**DOI:** 10.7759/cureus.6613

**Published:** 2020-01-09

**Authors:** Naseem Ahmed, Nadia Jawad, Saira Jafri, Wiky Raja

**Affiliations:** 1 Chest Medicine, Jinnah Postgraduate Medical Centre, Karachi, PAK; 2 Pulmonology, Jinnah Postgraduate Medical Centre, Karachi, PAK

**Keywords:** exacerbation, in-hospital mortality, decaf, curb-65, copd, aecopd

## Abstract

Objective

To compare the precision of DECAF (Dyspnea, Eosinopenia, Consolidation, Acidemia, Atrial Fibrillation) and CURB-65 scoring systems in prediction of mortality among patients presenting with an acute exacerbation of chronic obstructive pulmonary disease (COPD).

Material and methods

A prospective, cross-sectional study was done at the Department of Pulmonology, Jinnah Postgraduate Medical Centre, Karachi, Pakistan over a period of seven months, May 2019 through November 2019. Previously diagnosed patients of COPD (for more than six months), of either sex, aged between 40 and 70 years admitted primarily with an exacerbation were included in the study by non-probability consecutive sampling. Patients with myocardial infarction, chronic kidney disease and malignancy were excluded. All relevant data including patients’ demography, history, examination, DECAF and CURB-65 scores and in-hospital mortality were recorded on a proforma and later analyzed by using SPSS, version 20.0 (IBM Corp., Armonk, NY). Receiver operating characteristic (ROC) curve was drawn for comparison of accuracy of both scoring systems in prediction of in-hospital mortality (based on area under the curve (AUC)).

Results

There were 34 (29.8%) in-hospital mortalities while 80 (70.2%) survivals. AUC for DECAF score was 0.777 (0.673-0.881) and of CURB-65 was 0.715 (0.613-0.817) that reveals fair accuracy of the tests. Sensitivity of DECAF and CURB-65 scoring systems was almost similar i.e. 67.65% and 64.71% respectively, however DECAF was more specific than CURB-65 (86.25% compared to 68.75%, respectively).

Conclusion

The findings of our study suggest the use of a combination of scoring systems for prediction of in-hospital mortality in acute exacerbation of COPD based on appropriateness, access to facilities and clinician's preference.

## Introduction

Chronic obstructive pulmonary disease (COPD) represents a significant and increasing healthcare concern as a leading cause of morbidity and mortality, worldwide. The Global Burden of Disease Study reports a prevalence of 251 million cases of COPD globally in 2016 with an estimated 5% of all deaths being caused by the disease in 2015 [1]. Prevalence of COPD in Karachi, a cosmopolitan city of Pakistan, was reported to be 13.8% in 2014, which must have increased with the passage of time due to environmental pollution, poverty and low standard of living in the dense population of over 20 million in the city [2]. In a local study, a 23.6% prevalence of COPD was reported among asymptomatic and apparently healthy individuals [3].

An acute exacerbation of chronic obstructive pulmonary disease (AECOPD) is characterized by an episode of worsening respiratory symptoms (particularly dyspnea) requiring a change in medical treatment and/or hospitalization which may be life-threatening based on the severity of insult [4]. AECOPD accumulates increased morbidity, mortality and heavy socioeconomic burden due to hospitalization and intensive care.

The DECAF (Dyspnea, Eosinopenia, Consolidation, Acidemia, Atrial Fibrillation) score for prediction of inpatient mortality admitted with acute or repeated aggravation of disease is widely used and is a well-structured scoring system [5,6]. Acceptability of DECAF score is quite high as compared to BAP-65, CURB-65, CAPS, APACHE II risk scores in predicting in hospital mortality in AECOPD [7-10]. The most distinguishing trait of DECAF over the other scoring systems is its simplicity; as it is employed at bedside using indices of routine information on admission. In a large trial consisting of 2645 patients distributed into three cohorts, DECAF has shown consistently strong performance under the receiver operating curve (ROC) = 0.82-0.86, hence DECAF indices have been recommended for routine documentation on admission by COPD audit report 2014 of United Kingdom [11].

The CURB-65 score was derived and validated first time by Lim et al. based on 1068 patients from three prospective studies in the UK, New Zealand, and the Netherlands [12]. A 6-point score, one point for each of confusion, urea >7 mmol/l, respiratory rate ≥30/min, low systolic (<90 mm Hg) or diastolic (≤60 mm Hg) blood pressure, age ≥65 years (CURB-65 score) following initial hospital assessment data, enabled patients to be stratified according to increasing risk of mortality: score 0, 0.7%; score 1, 3.2%; score 2, 3%; score 3, 17%; score 4, 41.5% and score 5, 57%.

There were very few studies available for predicting the in-hospital mortality in AECOPD by comparison of CURB-65 with other indices particularly in local population. This study was designed to compare DECAF and CURB-65 as predictive indices of in-hospital mortality so that a management plan can be constructed soon after hospital admission by attending physicians according to the bed-capacity in ICU, available resources and treatment facilities which will help lessen the burden of morbidity and mortality in AECOPD.

## Materials and methods

This prospective cross-sectional study was conducted at the Department of Pulmonology, Jinnah Postgraduate Medical Centre, Karachi, Pakistan, after taking an approval from the institutional ethical review committee. Already diagnosed (on basis of history, exam, chest radiograph [CXR], spirometry) patients with COPD for more than six months, of either gender, aged between 40 and 70 years, who were admitted with primary diagnosis of AECOPD, were included in the study by non-probability consecutive sampling during May 2019 through November 2019. Signed informed consent was obtained from all enrolled patients or the attendant. Patients known to be suffering from acute myocardial infarction (MI) with a positive troponin-I, chronic kidney disease (CKD) with a creatinine of more than 1.5 mg/dl or malignancy were excluded.

The sample size was calculated using diagnostic accuracy calculator on the basis of sensitivity: 93%, specificity: 54%, margin of error for both: 12%, and prevalence: 0.40 for detecting mortality (score >2) [13]. The required sample size (n) was 114 patients with acute exacerbation of COPD.

Detailed history, thorough examination and relevant investigations were recorded. Both scores were calculated in all subjects. Effect modifiers like age, duration of COPD and comorbidities were controlled through strictly following inclusion, exclusion criteria and stratification.

DECAF has five variables: baseline dyspnea (eMRCD 5a or 5b), eosinopenia (<0.05 x 10^3^/dL), consolidation on chest radiograph, acidemia (pH < 7.30), atrial fibrillation and has a maximum score of 6. CURB-65 is a 5-point score, one point for each of: Confusion, urea >7 mmol/l, respiratory rate ≥30/min, low systolic (<90 mm Hg) or diastolic (≤60 mm Hg) blood pressure, age ≥65 years. At the time of discharge or death, the scores were compared for in-hospital mortality.

Statistical data were analyzed using SPSS, version 20.0 (IBM Corp., Armonk, NY). Numeric data including age, DECAF and CURB-65 scoring systems were explored for test of normality by using Kolmogorov-Smirnov test. Continuous data were presented by using descriptive statistics like mean, standard deviation, median and inter-quartile range (IQR). Wilcoxon-Mann-Whitney U test was employed to compare these numeric variables between in-hospital outcomes (survivals and expiries). Later, these variables were stratified. All categorical variables based on demographic characteristics, in-hospital outcome and positivity of DECAF and CURB-65 scoring systems were presented as frequencies and percentages. Chi-square test was performed to compare proportions of categorical variables between in-hospital outcomes. ROC curve was drawn for comparison of both scoring systems to predict in-hospital mortality, based on area under the curve (AUC). Sensitivity analysis was performed by using in-hospital mortality as gold-standard criterion. P-value ≤0.05 was considered statistically significant.

## Results

Out of 114 patients suffering from AECOPD, 76 (66.7%) were males and 38 (33.3%) were females (2:1). Exactly half of the patients were between 61 and 70 years of age followed by 51-60 years’ group (36%) and 35-50 years’ group (14%). About one-fourth of the patients were currently smokers, another one-fourth were non-smokers and half were ex-smokers.

There were 34 (29.8%) in-hospital mortalities in our study population, while 80 patients (70.2%) survived and were discharged from the hospital. Among 34 deceased, 25 (73.5%) were males and nine (26.5%) females. Among the survivors of in-hospital stay, 51 (63.8%) were males and 29 (63.8%) females. These data reveal insignificant difference of in-hospital outcomes between the genders (p = 0.311), age groups (p = 0.884) and smoking status (p = 0.515) (Table 1).

**Table 1:** Comparison of in-hospital mortality with demographic characteristics Numbers in parenthesis indicate percentages.

Factors	In-hospital outcome			P-value
	Total (n = 114)	Expired (n = 34)	Alive (n = 80)	
Gender				
Male	76 (66.7)	25 (73.5)	51 (63.8)	0.311
Female	38 (33.3)	9 (26.5)	29 (36.2)	
Age (Years)				
35–50	16 (14.0)	4 (11.8)	12 (15.0)	0.884
51–60	41 (36.0)	13 (38.2)	28 (35.0)	
61–70	57 (50.0)	17 (50.0)	40 (50.0)	
Smoking status				
Smoker	29 (25.4)	10 (29.4)	19 (23.8)	0.515
Ex-smoker	57 (50.0)	18 (52.9)	39 (48.8)	
Non-smoker	28 (24.6)	6 (17.6)	22 (27.5)	

Both scoring systems were analyzed in two different dimensions. In the first step, DECAF and CURB-65 scores were categorized into low-risk (i.e., scores 2 or below) and high-risk (scores >2) and compared for in-hospital outcomes.

Out of total 114 patients, 80 (70.2%) were low-risk in terms of DECAF score and 34 (29.8%) were high-risk. Among 34 patients who died in-hospital, 11 (32.4%) had low-risk and 23 (67.6%) had high-risk DECAF score while on the other hand those who survived the hospital stay i.e. 80, 69 (86.2%) had low-risk score and only 11 (13.8%) were high-risk. These data reveal significantly high proportions of patients with DECAF score>2 who expired during the hospital stay (p < 0.001) (Table 2).

For CURB-65 score, out of 114 patients, 67 (58.8%) were low-risk (score 2 or below) and 47 (41.2%) were high-risk (score >2). Out of 34 patients who died during the hospital stay, 12 (35.3%) were low-risk and 22 (64.7%) were high-risk. Fifty-five (68.8%) survivors had low-risk and 25 (31.2%) had high-risk CURB-65 scores. These data reveal significantly high proportions of patients with CURB-65 score >2 succumbing during the hospital stay (p < 0.001) (Table 2).

In the second step, DECAF and CURB-65 scores were analyzed into numeric expressions by using mean, standard deviation as well as median and interquartile range and compared for in-hospital outcomes. A similar pattern of statistical significance (p < 0.001) was observed (Table 2).

**Table 2:** Comparison of in-hospital mortality with DECAF and CURB65 scores ​​​​​​^a ^Significant difference in outcomes between high and low DECAF and CURB65 scores (Chi-square test). ^b ^Significant difference in outcomes between high and low DECAF and CURB65 scores (Mann-Whitney U test). SD: Standard deviation; IQR: Interquartile range.

Factors	In-hospital outcome			P-value
	Total (n = 114)	Expired (n = 34)	Alive (n = 80)	
Decaf (%)				
Negative (2 or below)	80 (70.2)	11 (32.4)	69 (86.2)	0.000^a^
Positive (above 2)	34 (29.8)	23 (67.6)	11 (13.8)	
CURB65 (%)				
Negative (2 or below)	67 (58.8)	12 (35.3)	55 (68.8)	0.001^a^
Positive (above 2)	47 (41.2)	22 (64.7)	25 (31.2)	
DECAF score				
Mean (SD)	2.10 (0.82)	2.71 (0.87)	1.84 (0.64)	0.000^b^
Median (IQR)	2 (1.25)	3 (1)	2 (1)	
Curb65 (%)				
Mean (SD)	2.20 (1.28)	2.88 (1.20)	1.91 (1.21)	0.000^b^
Median (IQR)	2 (2)	3 (2)	2 (2)	

**Figure 1:**
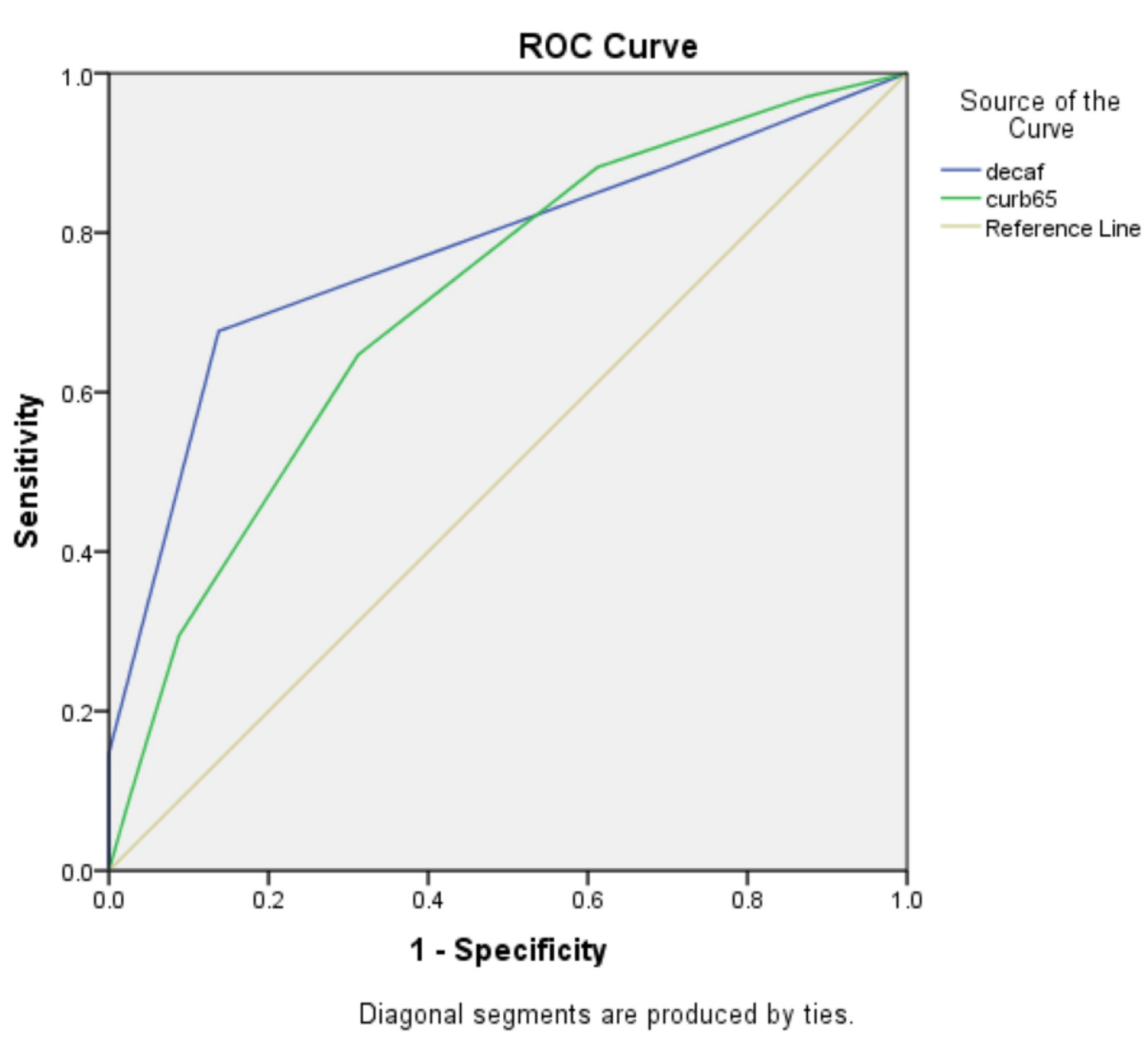
Receiver operating curve (ROC) for DECAF and CURB-65

AUC for DECAF score was 0.777 (0.673-0.881) and that of CURB-65 was 0.715 (0.613-0.817) that reveals fairly accurate tests (Table 3). The receiver operating curves (with AUC) for DECAF and CURB-65 are shown in Figure 1.

**Table 3:** Area under receiver operating curve of DECAF and CURB-65 scores AUC: Area under curve; SE: Standard error; CI: Confidence interval.

	AUC	SE	95% CI
DECAF	0.777	0.053	0.673–0.881
CURB-65	0.715	0.052	0.613–0.817

Sensitivity analyses have shown sensitivity, specificity, positive predictive value (PPV), negative predictive value (NPV) and accuracy of DECAF scoring system for prediction of in-hospital mortality respectively 67.65%, 86.25%, 67.65%, 86.25% and 80.7%. Sensitivity analyses of CURB-65 versus DECAF for prediction of in-hospital mortality have shown marginally less values of sensitivity, specificity, PPV, NPV and accuracy respectively 64.71%, 68.75%, 46.81%, 82.09% and 67.54% (Table 4).

**Table 4:** Area under curve of receiver operating curve of DECAF and CURB-65 CI: Confidence interval; NPV: Negative predictive value; PPV: Positive predictive value.

Tests	DECAF	CURB-65
Sensitivity (95% CI)	67.65% (49.47% to 82.61%)	64.71% (46.49% to 80.25%)
Specificity (95% CI)	86.25% (76.73% to 92.93%)	68.75% (57.41% to 78.65%)
PPV (95% CI)	67.65% (53.53% to 79.14%)	46.81% (36.89% to 56.98%)
NPV (95% CI)	86.25% (79.29% to 91.13%)	82.09% (73.96% to 88.09%)
Accuracy (95% CI)	80.70% (72.25% to 87.49%)	67.54% (58.14% to 76.01%)

## Discussion

The major outcome our study has shown is 34 (29.8%) in-hospital mortalities which was substantially higher than the mortalities reported in the validation studies of predicting in-hospital mortalities in acute ECOPD, i.e., 7.7% by Echevarria et al., 7.58% by Yousif and El Wahsh, 12.5% by Nafae et al., 17% by Kumar and Choubey, all of which had comparable sample sizes except the study by Echevarria et al. (sample of >800 patients) [6,8,13,14]. The reason for higher mortality rate in our study may be poverty, ignorance of the risk factors and symptoms of acute ECOPD and lack of awareness about emergency and routine healthcare services that an individual can have access to before their situation becomes life-threatening.

Multiple studies have documented the consistency and accuracy of the DECAF in foreseeing in-hospital mortality in AECOPD patients [5,6,10]. Similarly, our study has also shown good performance of the DECAF (AUC = 0.777). However, Yousif and El Wahsh concluded BAP-65 score to be better predictor than DECAF modified score [8]. Another retrospective study also showed CURB-65 was more sensitive (93.4% vs. 75.7%) than DECAF in predicting mortality [15].

On comparing the two scoring systems, the AUC for DECAF score was 0.777 (0.673-0.881) and for CURB-65 was 0.715 (0.613-0.817) that reveals fairly accurate results. The validation cohort confirmed a similar pattern [16]. DECAF appears to be a slightly stronger predictor of in-hospital mortality in AECOPD patients than the CURB-65. Similar studies that have compared the two scores have shown greater prognostic value of the DECAF in AECOPD. In these studies, AUC observed were 0.73 for CURB-65 (Chang et al.), 0.86 for DECAF and 0.66 (with consolidation)/0.72 (without consolidation) for CURB-65 (Steer et al.), 0.87 for DECAF and 0.65 for CURB-65 (Nafae et al.), which were fair enough (>0.80 is the acceptable AUC level worldwide for the validation of test and predictive indices) [5,13,17]. On the contrary, a study in Spain (Parras et al.) showed slightly greater accuracy of the CURB-65 as compared with DECAF (AUC 0.860 vs. 0.848) [15].

The CURB-65 score is a widely accepted tool to predict mortality risk in community-acquired pneumonia. Studies that have looked at the accuracy of CURB-65 (and other scores) in predicting 30-day mortality among patients admitted with pneumonia have shown high values for area under the receiver operating characteristic curve: AUC = 0.76 by Aujesky et al., AUC = 0.756 by Liu et al. and AUC = 0.835 by Shindo et al. [16,18,19].

Our study has shown that sensitivity of DECAF and CURB-65 scoring systems is almost similar: 67.65% and 64.71%, respectively. However, DECAF was more specific than CURB-65 (86.25% vs. 68.75%), which was similar to a study by Parras et al. [15]. Even after survival and hospital discharge, there may be high chances of recurrence of disease. As reported in a recent study, the rate of re-exacerbation in 90 days was 48.9% and was associated with certain clinical parameters in the current exacerbation as well as status before exacerbation [20].

A systematic review has identified several factors (related to demographics, comorbidities, acute physiologic derangements and COPD severity) associated with short (up to 90 days) and long (six months to two years) term mortality after admission. These factors may be used to develop new scores to accurately predict in-hospital death and outcomes after discharge and thus help clinical decision-making with regard to appropriate level of care and safe discharge [7]. Our study is limited in the sense that we have not assessed the various components of the two scores separately. Individual analysis of these and other factors could lead to formulation of newer scores which may prove to be still better predictors of mortality in this group of patients.

## Conclusions

We suggest the use of both scoring systems for risk stratification of AECOPD patients according to clinicians’ preference and access to basic assessment tools in a resource-poor country like ours. However, the DECAF has shown superior performance as compared with the CURB-65 in terms of being a predictor of in-hospital mortality and thus guiding physicians about timely escalation of treatment among high-risk groups.
